# Factors Associated With Low Tuberculosis Case Detection and Investigation in Public Health Facilities (PHFs) in Bududa District, Uganda: A Cross‐Sectional Study

**DOI:** 10.1002/hsr2.70438

**Published:** 2025-02-10

**Authors:** Imelda Tumuhairwe, Alimah Komuhangi, Alfred Okello, Apolo Ayebale, Ambrose Wabwire Buyinza, Godfrey Bwire

**Affiliations:** ^1^ Department of Health Dududa District Local Government Bududa Uganda; ^2^ Institute of Public Health and Management Clarke International University Kampala Uganda; ^3^ Ahaki, Afya na Haki Institute Kampala Uganda; ^4^ Department of Health Amuru District Local Government Amuru Uganda; ^5^ Department of Integrated Epidemiology Surveillance and Public Health Emergencies, Ministry of Health Kampala Uganda; ^6^ Department of Architecture and Physical Planning Makerere University Kampala Uganda; ^7^ Department of Community Health and Behavioral Sciences, School of Public Health Makerere University Kampala Uganda

**Keywords:** Africa, epidemiology, infectious diseases, public health, tuberculosis, Uganda

## Abstract

**Background and Aims:**

Despite global efforts to combat tuberculosis (TB), Uganda bears a high burden, with an annual incidence of 200 per 100,000 and a mortality rate of 35 per 100,000 persons. This study investigates TB prevalence, detection rates, and associated factors in public health facilities (PHFs) in Bududa district.

**Methods:**

A cross‐sectional study employing Mixed Methods Research (MMR) was conducted in November 2019 on respondents with symptoms suggestive of TB and key informants overseeing TB care in Bududa district, Uganda.

**Results:**

Only 18.8% (46/245) of respondents who reported symptoms suggestive of TB were investigated for infection. The majority, 87.4%, never had sputum requested and 91.7% never had a chest X‐ray done. Participants from rural areas were 26% less likely to be screened for TB than their urban counterparts (PR = 1.26, 95% CI [1.16–1.38]). Challenges for TB care included staffing shortages and inadequate medical supplies and equipment.

**Conclusion:**

This study highlights the disparity between high TB burden and low detection rates in Bududa district. The Government of Uganda and stakeholders should invest in solving the challenges identified by this study.

## 1 Introduction

Although great strides have been made to control and cure tuberculosis (TB), many people still get sick and die from this disease. Globally, an estimated 10.6 million people fell ill with TB in 2021 [1], out of whom 1.6 million deaths were reported [2]. TB is a preventable and treatable infectious disease caused by a bacteria, *Mycobacterium tuberculosis*. Not everyone infected with TB bacteria becomes sick. Consequently, there are two manifestations of TB, namely, latent TB infection (LTBI) and TB disease [3]. TB is commonly spread from one person to another through the air by coughing, sneezing, and speaking [4]. Patients with TB present with cough, fever, night sweats, weight loss, and appetite loss [1]. Diagnosis of TB in the majority of the suspected cases is done by sputum smear microscopy to identify the acid‐fast bacilli [5]. Due to advances in diagnostics, there are new tests that give more reliable results quickly, namely, the GeneXpert *M. tuberculosis*/Resistance to Rifampin Assay (MTB/RIF assay), which gives reliable results within 2 h [6].

In 2023, TB prevalence per 100,000 of the population was highest in Africa (226), followed by Southeast Asia (116), Eastern Mediterranean (114), West Pacific (77), the Americas (29) and Europe (25) [7]. Most of the cases in Africa are reported from sub‐Saharan Africa from countries with high levels of poverty, HIV/AIDs, or both [8]. TB is a major cause of morbidity and mortality in Uganda, with an estimated incidence of 200 per 100,000 persons and a mortality rate of 35 per 100,000 persons [9, 10].

TB treatment plays a vital role in controlling the infection, preventing the progression of the disease, and protecting both personal and community health [11]. Hence, early detection and treatment of cases are very important for TB infection control and, ultimately, TB elimination [12]. For TB cases to be identified and treated, cost has to be encored by the government or the individuals in the form of direct or indirect medical and nonmedical costs [10]. In terms of the affected countries, Uganda is one of the 30 countries with a high TB burden as designated by the World Health Organization (WHO). Within Uganda, the infection is more common among HIV/AIDS patients and persons living in crowded areas [13]. In 2015, the Ministry of Health carried out a TB survey and found that the prevalence of TB was 259 and far higher than the reported number of 159/100,000 persons, which was equivalent to 39% of TB infections not diagnosed [14]. The factors responsible for missed TB diagnosis were long distances, high cost of seeking care, self‐treatment, and ignored illness [15, 16]. To address the high TB burden, the Government of Uganda through the Uganda National Tuberculosis and Leprosy Control Program promotes prompt screening for TB among all persons seeking health care at healthcare facilities (private or government) [17]. Prevention of TB is also affected by inadequate knowledge and skills among health workers; lack of laboratory diagnostics; and low staffing levels, medical supplies, and infrastructure [18]. A study in a rural district of Eastern Uganda identified a lack of motivated and dedicated TB focal persons, inadequate funding for TB activities, and poor implementation of community Direct Observed Treatment [19].

Private health facilities, mainly for‐profit ones, deliver most of the health services in Uganda [20]. However, most of these private facilities have limited or nonexistent involvement in TB diagnosis due to a lack of capacity [15]. A study conducted in Wakiso district, central Uganda, identified individual characteristics such as age, sex, educational level, and distance from health facilities to affect whether one gets investigated or not [21]. The prevalence and factors associated with a low TB detection rate in Bududa district are not known. This study aimed to determine the proportion of patients who had symptoms that might suggest TB and who received care and TB testing at public health facilities (PHFs) in Bududa district and to explore the health facility factors that were associated with this proportion. The findings will guide TB prevention and treatment in Bududa districts and districts with similar rural settings in Uganda.

## 2 Materials and Methods

### 2.1 Study Design

This was a cross‐sectional study carried out using quantitative and qualitative data collection techniques (MMR). The quantitative aspect determined the proportion of participants with symptoms suggestive of TB who were investigated, while the qualitative part provided more information on the factors associated with missed opportunities in TB investigation in PHFs in Bududa district.

### 2.2 Study Setting

**Figure 1:**
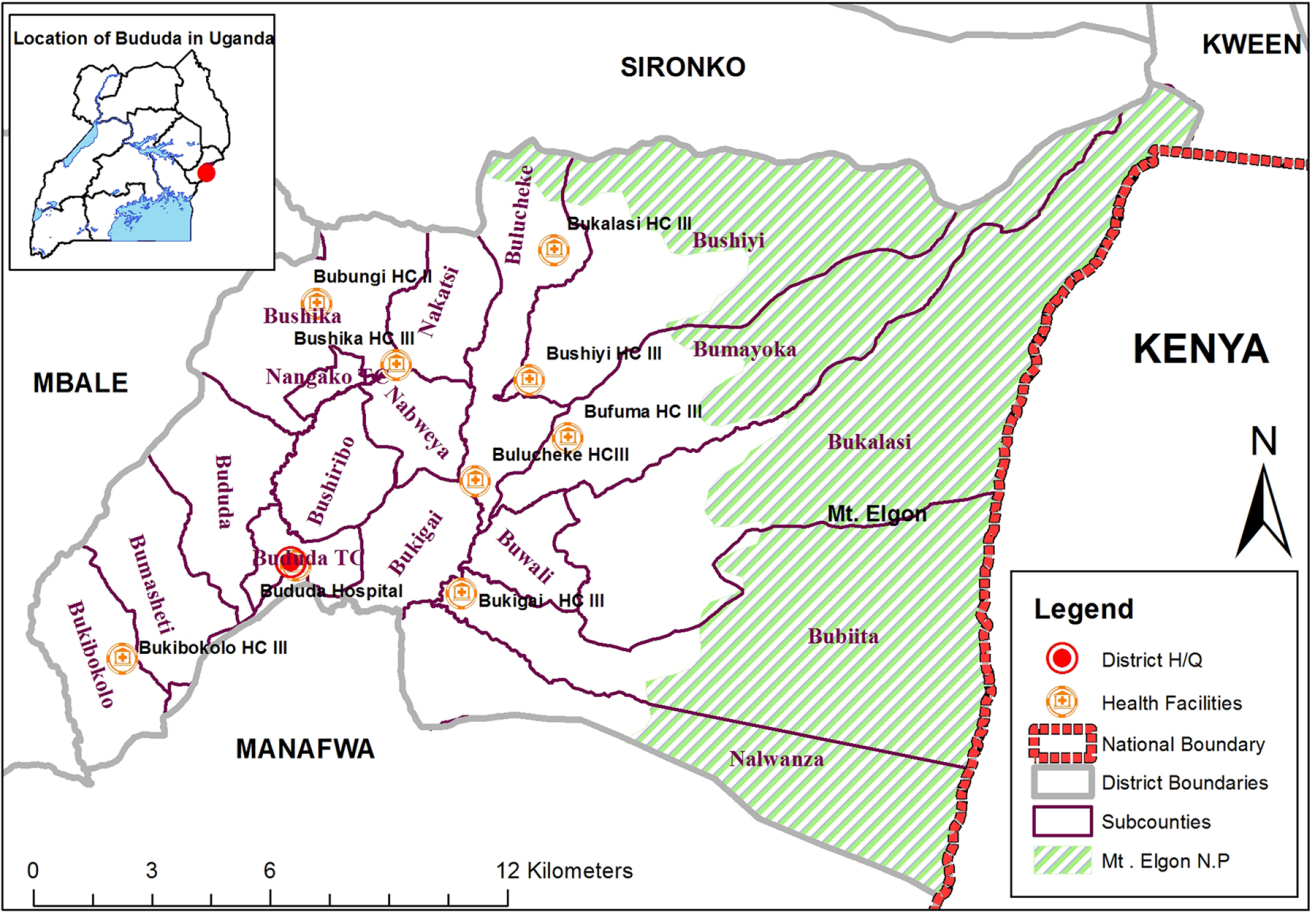
Map of Bududa district showing the administrative areas and the location of the public health facilities that were studied. The round brown circles with a cross represent the health facilities that were studied. The green strip is the area covered by Mt. Elgon National Park.

The study was conducted in Bududa district found in Eastern Uganda on the western slopes of Mt. Elgon. Bududa district is bordered by Sironko district to the north, Kenya to the east, Manafwa district to the south, and Mbale district to the west. The population of Bududa district was estimated at 259,800 in 2019 with annual population growth rate of 4.5% [22]. The locations of Bududa district in Uganda and the PHFs are shown in Figure 1.

### 2.3 Study Population

The study enrolled adult persons presenting with signs and symptoms suggestive of TB (cough, evening fevers, night sweats, and weight loss) for more than 2 weeks and HIV‐positive patients with signs and symptoms of TB or a cough for more than 24 h, seen at OPD and HIV clinic of the nine PHFs that offer TB diagnostic services and ART in Bududa district.

### 2.4 Inclusion and Exclusion Criteria

Persons aged 18–54 years for males and 18–49 years for females with signs and symptoms suggestive of TB (cough for more than 2 weeks, weight loss, night sweats, and evening fevers for HIV‐negative patients, and a cough for more than 24 h in HIV‐positive patients) who presented in OPD and HIV clinic of the PHFs and were able to provide written consent for participation in the study were included. On the other hand, persons in the above age group (male or female) were excluded if they had sent their sputum for investigation before the visit, were known as TB patients on treatment, and were the persons who were severely sick to respond to questions.

### 2.5 Sample Size Determination

The sample size was determined as previously described by Kish and Leslie [23].

n=Z2Pq/d2

where *n* = sample size, *z* = the standard normal value corresponding to 95% confidence interval (1.96), *P* = proportion of patients with symptoms and signs suggestive of TB investigated for TB and *q* = 100% − *P* or (1 − *P*). Previous studies conducted in Uganda showed that TB prevalence among those investigated (*P*) was 21.9% [24].

Substituting to find *q*, *q* = 100% − *P* or (1 − *P*), which was 1 − 0.21 = 0.79;

*d* = margin of error (0.05) at 95% CI.

Substituting in the formula, *n* = *Z*2*Pq/d*2; the required number of patients,

*n* = 1.962 × 0.21 × 0.79/0.052 = *n* = 255 patients.

### 2.6 Selection of Study Participants

Purposive enrollment of participants in OPD and ART clinic was done as they exited the clinician's room. The patients meeting inclusion criteria were briefed by the trained research assistants (RAs) on the study and the purpose. The consenting patients were then recruited, offered written consent, and interviewed using a questionnaire. The number of patients interviewed in each facility was determined according to probability proportionate to facility size, and this depended on the daily OPD attendance. A simple random sample of respondents was selected from each PHF using a coin toss method. The target number of respondents for each PHF was set according to daily attendance. All attendees at each PHF who met the inclusion criteria were given an equal chance of being selected by flipping a coin, with heads indicating enrollment into the study and tails indicating not enrolled. The process was repeated until the target number of respondents for each facility was reached (Table 1).

**Table 1:** The OPD attendance and the number of participants enrolled for each of the nine health facilities in Bududa district with TB diagnostics services.

Facility	Average OPD attendance	% of Total OPD attendance	Targeted no. of presumptive TB
Bududa hospital	204	28.0	72
Bukigai HCIII	66	9.0	23
Bushika HCIII	71	9.7	24
Bukibokoro HCIII	55	7.5	19
Bubungi HCIII	22	3.0	7
Bukalasi HCIII	120	16.4	42
Bulucheke HCIII	85	11.6	30
Bufuma HCIII	58	7.9	20
Bushiyi HCIII	50	6.9	18
Total	731	100	255

### 2.7 Data Collection

Data were collected by the RAs who were identified and trained for 2 days to understand the study protocol and the methods of work before they commenced data collection. Throughout the process, supervision of RAs was done by the lead author. For quantitative data, the RAs enrolled participants as they exited the examination rooms. Participants who met inclusion criteria and consented to participate in the study had a questionnaire administered to them to find out whether they were asked to provide sputum, a chest X‐ray (CXR), or samples for GeneXpert. Data collected on the participants included the age, sex, place of residence, education level, occupation, entry point and distance to a health facility, the reason for facility visit, reporting symptoms to the examining clinician, HIV status, clinicians asking symptoms and signs for TB, referral status, presence of signs and symptoms (cough, fever, weight loss, and night sweats), and duration of symptoms. Those participants with signs and symptoms suggestive of TB who were found not investigated for TB were noted and referred back to the examination rooms for appropriate TB investigations. Data were also collected in all the nine facilities by use of an observational checklist to identify facility factors associated with TB missed opportunities such as staffing levels, availability of medical supplies, sputum containers, availability of TB medicines and supplies, availability of TB reagents, presence of technical staff, availability of standard TB diagnostic equipment (microscope, CXR machine, and GeneXpert), and availability of TB guidelines.

To obtain qualitative data, 21 key informants were interviewed by trained RAs aided by a pre‐tested key informant guide. Two key informants were selected from each of the nine health facilities (the head of the facility and the focal person for TB prevention in the facility). Additionally, one person from the district and two others, one from each of the two health sub‐districts, were purposively selected and interviewed. These individuals were chosen based on their involvement in providing care and treatment for TB and ART.

### 2.8 Data Management

Data were coded and double‐entered into Microsoft Excel and STATA (Version 13) [25] for storage and analysis. Data were cleaned every day after the interviews and backed up. Initial data analyses were done in Excel and Stata Version 13. Subsequently, the dataset was imported into Statistical Package for Social Sciences (SPSS) Version 12 for advanced statistical modeling. In SPSS, Bonferroni correction was utilized to account for multiple comparisons, mitigating the risk of false positives and enhancing the validity of results. Data were analyzed to get the frequencies, means, percentages, *p* values, and prevalence ratios and presented in tables. The categorical variables were analyzed using *χ* ^2^ test. *p* < 0.005 was considered significant. The association of factors was done through multivariate analyses by a modified Poisson regression model, and adjusted prevalence ratios and 95% confidence intervals were estimated. Key informant interviews were transcribed, creating overarching categories and themes. The findings were presented in the form of text or quotes.

### 2.9 Ethical Consideration

Clearance to conduct this study was sought from the Research Ethics Committee of Clarke International University, CIUREC/0177; Uganda National Council of Science and Technology, UG‐REC‐015; and Bududa district local administration. Training of the research team on the study protocol and ethical principles was undertaken before data collection. Written informed consent was requested from all participants before their enrollment into the study. A copy of the consent form was given to all consenting participants. Confidentiality was observed throughout the study by conducting individual interviews and using unique identification numbers instead of names. Stored data were only accessible to the study investigators. Where necessary, permission was obtained from the participants to audio record the interview. If the participant's permission was not granted, the researchers took extensive notes of the discussion. Interview transcripts were only used for this study and destroyed thereafter.

## 3 Results

### 3.1 Demographic Characteristics

A response rate of 97.6% (249/255) was recorded. The respondents were aged 18–54 years (mean = 34.1; SD = 10.2 years). The majority of the respondents were female 78.3% (195/249), and most of them (77.1%; 192/249) were identified from the OPD. Most 96.4% (240/249) of the respondents were from rural areas and were staying beyond 1 km from the healthcare facility 78% (199/249). The majority, 91.8% (234/249), of the respondents had attained at least primary school education. Demographic characteristics of the respondents are shown in Table 2.

**Table 2:** Demographic characteristics of study participants.

Characteristic	Category	Frequency	Measure
Age in years			
	< 30	87	34.9%
	30–44	111	44.6%
	≥ 45	51	20.5%
	Mean (SD)		34.1 (10.2)
Sex			
	Males	54	21.7%
	Females	195	78.3%
Employment			
	Employed	25	10.0%
	Unemployed	47	18.9%
	Peasant farmer	177	71.1%
Entry point			
	ART Clinic	57	22.9%
	OPD	192	77.1%
Education level			
	None	19	7.6%
	Primary	179	71.9%
	Secondary and higher	51	20.5%
Distance from facility			
	< 1 km	54	21.7%
	1–5 km	154	61.9%
	> 5 km	41	16.4%
Marital status			
	Yes	183	73.5%
	No	66	26.5%
Residence			
	Urban	9	3.6%
	Rural	240	96.4%

### 3.2 Proportion of the Patients Investigated for TB

Only a small proportion, 18.8% (46/256), of respondents (patients) who reported having had symptoms suggestive of TB were investigated for TB. The majority of the respondents, 81.2% (199/245), reported having had symptoms suggestive of TB but had not been screened for TB by having their sputum tested or getting a CXR. For example, 87.4% (214/245) of the patients never had sputum requested, and 91.7% (222/242) never had CXR done. Patients who came from rural areas were 26% more likely to miss TB investigation compared to those from urban areas (PR = 1.26, 95% CI [1.16–1.38]). The proportion of patients who missed TB screening in PHFs is shown in Table 3.

**Table 3:** The proportion of respondents who missed TB screening by point of entry into the facility and their demographic characteristics in PHFs in Bududa district.

Individual factors	Missed TB screening		Unadjusted model	*p* value	Adjusted model
			PR (95% CI)		PR (95% CI)
	Yes (*n* = 199)	No (*n* = 46)			
Entry point					
ART clinic	39 (70.91)	16 (29.09)	1		1
OPD	160 (84.21)	30 (15.79)	0.89 (0.80–0.99)*	0.03	0.88 (0.79–0.98)*
Sex					
Female	154 (80.63)	37 (19.37)	Reference		
Male	45 (83.33)	9 (16.67)	0.98 (0.89–1.08)	0.65	
Education					
None	15 (78.95)	4 (21.05)	Reference		
Primary	144 (82.29)	31 (17.71)	0.99 (0.85–1.15)		
Secondary or higher	33 (78.57)	9 (21.43)	1.03 (0.86–1.23)	0.93	
Employment					
Employed	19 (76.00)	6 (24.00)	Reference		
Unemployed	33 (71.74)	13 (28.26)	1.03 (0.87–1.22)	0.11	
Peasant farmer	147 (84.48)	27 (15.52)	0.93 (0.80–1.07)		
Distance from facility					
< 1 km	44 (81.48)	10 (18.52)	Reference		
1–5 km	125 (82.24)	27 (17.76)	0.99 (0.89–1.10)	0.75	—
> 5 km	30 (76.92)	9 (23.08)	1.03 (0.90–1.19)		
Residence					
Urban	9 (100.00)	0 (0.00)	1		1
Rural	190 (80.51)	46 (19.49)	1.19 (1.14–1.24)**	0.14	1.26 (1.16–1.38)**
Referral					
No	180 (81.08)	42 (18.92)	Reference		
Yes	12 (80.00)	03 (20.00_	1.00 (0.84–1.20)	0.92	

*Note:* There is a significant association between explanatory variables and respondents reporting to have missed TB screening or investigation.

Abbreviations: CI = confidence interval; PR = prevalence ratios.

** *p* < 0.001

* *p* < 0.05

### 3.3 Clinical Manifestation of Patients

Study participants presenting with weight loss were 23% less likely to miss investigation and screening for TB than those without weight loss (PR = 0.77, 95% CI [0.67–0.88]). The proportion of respondents who missed TB screening or investigation by the presenting clinical features is shown in Table 4.

**Table 4:** Percentage of respondents who missed TB screening.

Clinical factors	Missed TB screening or investigation		Unadjusted model	*p* value	Adjusted model
			PR (95% CI)		PR (95% CI)
Symptoms	Yes (*n* = 199)	No (*n* = 46)			
Cough					
No	2 (100)	0 (100)	Reference		
Yes	197 (81.07)	46 (18.93)	1.18 (1.14–1.23)***	0.50	
Persistent fever					
No	7 (87.50)	1 (12.50)	Reference		
Yes	192 (81.36)	44 (18.64)	1.05 (0.85–1.29)	0.66	
Night sweats					
No	13 (92.86)	1 (7.14)	1		
Yes	185 (80.43)	45 (19.57)	1.11 (0.97–1.27)	0.25	
Weight loss					
No	34 (66.67)	17 (33.33)	1		
Yes	165 (85.49)	28 (14.51)	0.85 (0.77–0.95)**	0.002	0.77 (0.67–0.88)***
Reporting all symptoms					
Yes	147 (84.00)	28 (16.00)	Reference		
No	52 (74.29)	18 (25.71)	1.08 (0.98–1.19)	0.08	0.89 (0.80–0.99)*
Duration of symptoms					
< 2 weeks	1 (100.0)	0 (0.00)	Reference		
2 weeks to 1 month	110 (81.48)	25 (18.52)	1.18 (1.12–1.25)***	0.88	
> 1 month	88 (80.73)	21 (19.27)	1.19 (1.12–1.26)***		
Asked about symptoms					
No	97 (87.39)	14 (12.61)	Reference		
Yes	102 (76.12)	32 (23.88)	1.10 (1.01–1.19)*	0.03	1.10 (1.02–1.19)**
Reported symptoms					
No	64 (82.05)	14 (17.95)	Reference		
Yes	127 (79.87)	32 (20.13)	1.01 (0.93–1.11)	0.69	
Knows HIV status					
No	49 (87.50)	7 (12.50)	Reference		
Yes	149 (79.26)	39 (20.74)	0.17		

*Note:* There is a significant association between explanatory variables and respondents reporting to have missed TB screening or investigation.

Abbreviations: CI = confidence interval; PR = prevalence ratios.

*** *p* < 0.001

** *p* < 0.01

* *p* < 0.05.

### 3.4 Staffing Level and Availability of Medical Supplies and Equipment for TB Screening/Diagnosis

All facilities had registers, guidelines, and TB screening in the outpatient department. Status of availability of TB screening resources is given in Table 5.

**Table 5:** Status of TB screening resources in PHFs in Bududa districts during the study period.

Facility characteristic	Frequency (*n*)	Percentage (%)
TB register available	9	100.0
Presumptive TB register available	9	100.0
Facility has TB guidelines	9	100.0
TB diagnosis desk Aids available	9	100.0
Investigation Case Finding (ICF) forms available	8	77.8
TB screening at the OPD entry point	9	100.0
TB screening at the HIV clinic entry point	8	88.9
Supplies available	8	88.9
Functional microscope	8	88.9
GeneXpert machine	1	11.1
Functional CXR machine	0	0.0
TB focal person	9	100.0
Functional TB clinic	8	88.9
Anti‐TBs in stock	8	88.9
Health workers at the facility that offer care to TB clients		
1–3 workers	6	66.7
4–7 workers	2	22.2
> 7 workers	1	11.1
Average number of clients' attendance per day		
˂ 30 clients	3	33.3
31–60 clients	5	55.6
> 60 clients	1	11.1
Average number of TB clients diagnosed monthly		
1–3 clients	8	88.9
> 3 clients	1	11.1

The facility factors significantly associated with missing TB screening or investigation included the number of TB patients diagnosed monthly and screening for TB in the ART clinic. Participants attending PHFs diagnosing more than three TB cases per month were 16% less likely to miss TB screening or investigation compared to those diagnosing less than three TB cases per month (PR = 0.84, 95% CI [0.76–0.93]). Participants attending facilities where screening for TB is done at the ART clinic entry points were 13% more likely to miss TB screening than those not (PR = 1.13, 95% CI [1.01–1.27]).

### 3.5 Barriers Health Workers Face in Investigating TB in PHFs in Bududa District

Key informants were asked to share what they thought hindered health workers from carrying out TB investigations and diagnoses. The respondents stated that the patient factors that hindered health workers from carrying out TB investigations and diagnoses were the reluctance of the patients to test and their limited knowledge of TB. On the other hand, the health facility factors, the key informant respondents noted, were high workload, lack of technical staff to carry out TB investigation and diagnosis, lack of enough protective gears, stockouts for TB drugs, lack of electricity, and equipment breakdown. The key informants also noted that the fear of contracting TB, poor attitudes of the workers, and poor motivation of the health workers hindered TB investigation and diagnosis. One of the key informants had this to say;

…. first of all, on the side of patients, what greatly hinders TB investigation and diagnosis is that most of them fear to test for TB while others do not have the knowledge on what TB is and where to get medication if found with TB.

On the other hand, the factors that hinder health workers from carrying out TB investigation and diagnosis vary from the lack of equipment for TB testing, lack of trained personnel to carry out the tests and most importantly some of them fear that they might also contract TB during the process of investigating and diagnosing TB since they lack protective gears…

There were also respondents who referred to the guidelines. One key informant said,

…. well, the guidelines for TB screening and diagnosis are that those who cough for 2 weeks have to be screened for TB. Thereafter, sputum is taken for testing in the lab. Those tested positive are initiated on treatment and those who test negative are taught how to prevent themselves from acquiring TB….

## 4 Discussion

This study shows that there is low TB case detection in PHFs in Bududa district, with a prevalence of 81.2% (199/245). The factors responsible for this low case detection rate are varied and include those related to the health facilities and those from the patients seeking care. The health facility factors identified included high workload, lack of protective gear, medicine stockouts, equipment breakdown and power shortages, fear of contacting TB by the Health workers, negative attitude of some health workers, and low motivation of staff to manage TB cases. Consistent with previous studies, this study highlights the critical challenges posed by heavy health worker workloads and technical staff shortages in effective TB investigation and diagnosis [26]. Health workers may be overburdened with other responsibilities, which can lead to delays in TB diagnosis and treatment [26]. Similar findings where there is low TB detection in rural PHFs were recorded in other countries in Africa such as Ghana and Ethiopia. The reasons for low TB detection rate in Ghana and Ethiopia were poor understanding of TB and its symptoms, poor knowledge of where to seek care, and poor health service infrastructure with limited outreach services [27, 28]. Hence, to address this issue of low TB detection in Bududa district and other districts in rural Uganda, it may be helpful to increase staffing levels and provide additional training to health workers to improve their capacity to diagnose and treat TB.

Lack of diagnostic equipment, protective gear, and stock‐outs of TB medicines can also be significant barriers to effective TB investigation and diagnosis [29, 30, 31]. The characteristics of the facilities visited can determine whether one gets their sputum taken or CXR done. For instance, there were no functional CXR equipment in PHFs studied, including in Bududa hospital, which is expected to have X‐ray and GeneXpert machines. Health centers without these services were, therefore, expected to have a much lower TB detection rate than Bududa hospital with GeneXpert machine. Also, one of the facilities had no TB medicines during the study period. Health workers may be reluctant to diagnose and treat TB patients if they do not have access to the necessary protective gear or medicine. Hence, there is a need to equip the PHFs with the required TB diagnostic equipment, protective gear, TB medicines, and supplies.

Among the respondents, patient factors such as lack of awareness and low TB knowledge were also noted. This low‐level awareness could have contributed to a low TB detection rate in a similar manner as reported in a previous study [32]. In addition to the health facility‐related factors, this study identified patient‐related factors such as reluctance of the patients to test for TB and low knowledge of TB. Reluctance of patients to test for TB can be a major barrier to early diagnosis and treatment [15]. This reluctance may stem from a variety of factors, including fear of stigma, lack of awareness about TB, and concerns about the cost of testing and treatment [33, 34, 35]. To address this issue, it may be helpful to increase community awareness about TB and the importance of early diagnosis and treatment. This could involve targeted education campaigns, community outreach programs, and other initiatives to engage patients and their families.

This study found that the detection rate of TB in Bududa district (Eastern Uganda, rural district) was lower than in Wakiso district (Central Uganda, urban district) and in South Africa [16, 36]. The higher detection rate in Wakiso district could be due to Wakiso being in Central Uganda, near the Kampala capital city, and because it had more health workers with better equipment. The same reason could apply to South Africa, where better development indices could contribute to the observed difference. Therefore, addressing the staffing levels and health facility factors identified in this study should be able to alleviate TB detection rates in Bududa district. We also think that the factors contributing to low TB detection and investigation may not be limited to Bududa districts alone but may also be present in the neighboring districts that share similar physical and geographical features, such as mountain terrains, landslides, and rural settings. Hence, these findings could be used by the Uganda Ministry of Health to improve TB detection, investigation, and treatment in Bududa districts and the neighboring districts.

### 4.1 Strength and Study Limitations

This study's primary strength stems from its utilization of a Mixed Methods Research (MMR) approach (combining qualitative and quantitative techniques), which facilitated a rigorous and comprehensive examination of the factors underlying the alarmingly low TB detection rates in Bududa district. While the study has strengths of MMR, there were limitations due to its cross‐sectional design. Consequently, causal relationships between associated factors and low TB detection levels could not be established. Furthermore, we only studied respondents in OPD and ART clinics, excluding the inpatient department, which cares for more severely ill TB cases. To comprehensively understand factors related to TB in PHFs in Bududa district, future studies using prospective cohorts or other rigorous study designs and data analyses are needed. Another limitation of this study's findings is that we relied on patient‐reported data for TB symptoms, medical visits, and diagnostic tests, which could have introduced recall bias. Although the use of a standardized questionnaire mitigated this risk, validating patient‐reported information through objective measures (e.g., point‐of‐care record cross‐checks and targeted patient verification) is recommended for similar future research. Likewise, though this study offers valuable insights into rural Uganda, its external validity is limited by Bududa district's unique socioeconomic, geographical, and cultural characteristics. Therefore, caution is warranted when extrapolating findings to urban areas or diverse international contexts, where different factors may shape outcomes. To enhance generalizability, future research should investigate the applicability of these findings across varied settings. Nevertheless, this study adds to the growing body of evidence of the factors associated with low TB detection rates in the PHFs in Bududa and Uganda in general.

## 5 Conclusion

This study reveals a high TB prevalence and low detection rate in Bududa district. To achieve the United Nations' goal of ending the global TB epidemic by 2035, Uganda must urgently invest in strengthening healthcare capacity of PHFs; addressing gaps in staff, equipment, protective gear, TB medicines, and supplies; and ensuring reliable electricity in Bududa district and other rural districts with high TB prevalence. Future research on TB in Bududa district could build upon this study to better inform targeted TB interventions in Bududa and similar TB high‐prevalence areas.

## Author Contributions

**Imelda Tumuhairwe:** conceptualization, writing–original draft, data curation, formal analysis, methodology, investigation, supervision, writing–review and editing, visualization, validation. **Alimah Komuhangi:** conceptualization, supervision, writing–review and editing, writing–original draft, methodology, validation. **Alfred Okello:** formal analysis, data curation, investigation, writing–review and editing, validation, visualization, writing–original draft. **Apolo Ayebale:** formal analysis, writing–review and editing, investigation, validation, data curation, visualization, writing–original draft. **Ambrose Wabwire Buyinza:** methodology, data curation, validation, formal analysis, writing–review and editing, visualization, writing–original draft. **Godfrey Bwire:** conceptualization, supervision, writing–review and editing, writing–original draft, methodology, validation.

## Conflicts of Interest

The authors declare no conflict of interest.

### Transparency Statement

The lead author Imelda Tumuhairwe affirms that this manuscript is an honest, accurate, and transparent account of the study being reported; that no important aspects of the study have been omitted; and that any discrepancies from the study as planned (and, if relevant, registered) have been explained.

## Data Availability

The data that support the findings of this study are available on request from the corresponding author. The data are not publicly available due to privacy or ethical restrictions.
